# Writing creativity, cohesion, and formal linguistic competence in LLMs: A comparative evaluation based on English and Chinese continuation writing

**DOI:** 10.1371/journal.pone.0335185

**Published:** 2026-06-22

**Authors:** Yao Zhang, Tongquan Zhou, Yulu Li, Yizhong Xu, Taohui Li, Juan Wang

**Affiliations:** 1 School of Foreign Languages, Southeast University, Nanjing, China; 2 College of Chinese Literature and Language, Qufu Normal University, Qufu, China; 3 College of Foreign Languages, Nanjing University of Aeronautics and Astronautics, Nanjing, China; 4 College of Foreign Languages, Qufu Normal University, Qufu, China; National University of Malaysia Faculty of Education: Universiti Kebangsaan Malaysia Fakulti Pendidikan, MALAYSIA

## Abstract

Recent advancements in large language models (LLMs), such as GPT-3.5, GPT-4, and Chinese models like ERNIE and SparkDesk (SPARK), have shown remarkable potential in story generation. However, a direct comparison between ChatGPTs, ERNIE, and SPARK (pretrained with dominant Chinese Corpus, referred to herein as C-dominant LLMs) remains underexplored in this domain, particularly regarding narrative writing continuation ability. Identifying such difference would be beneficial to teachers to adopt LLMs in writing practice. This study addresses this issue by comparing ChatGPTs and C-dominant LLMs in story continuation tasks, with a focus on cohesion, creativity, and formal linguistic competence. We harnessed advanced analytical tools, including Coh-Metrix and CTAP for cohesion and LIWC-22 and CLIWC for creativity, to assess the performance of GPT-3.5, GPT-4, ERNIE, and SPARK across English and Chinese continuations. Results indicated that C-dominant LLMs surpassed ChatGPTs in referential and deep cohesion for English story generation, while ChatGPTs excelled in latent semantic analysis. Conversely, ChatGPTs demonstrated superior performance in cohesion across all metrics for Chinese story generation. In terms of creativity, GPT models behaved better in image for English texts and voice for both English and Chinese, whereas C-dominant LLMs excelled in image for Chinese continuations. For formal linguistic competence, GPTs showed better performance in English tasks, while C-dominant LLMs in Chinese tasks. These findings provide insights for optimizing LLMs in multilingual contexts and offer guidance for their application in writing and translation based on diverse languages.

## Introduction

The release of ChatGPT by OpenAI in November 2022 has aroused explosive enthusiasm towards large language models (LLMs) around the world. As a response to the phenomenon, a couple of powerful Chinese AI companies also launched their LLMs, among which were the representatives ERNIE (Enhanced Representation through kNowledge IntEgration) bot [1] and SparkDesk [2]. These LLMs were pretrained primarily based on the Chinese corpus, compared with GPT-3.5 and GPT-4. Therefore, in the following sections, the ERNIE and SPARK will be collectively mentioned as C-dominant LLMs, while GPT-3.5 and GPT-4 are referred to as ChatGPTs. The four LLMs were designed to respond to users’ requests with appropriate outputs in natural language [3] and are able to finish natural language tasks and generate complicated texts [4,5]. Therefore, it can be inferred that LLMs are capable of creating reasonable and logical continuing story based on given texts. However, currently no research has investigated and compared the story continuing ability of LLMs. Understanding the differences between LLMs would be of great benefit for choosing and applying best models to narrative teaching and learning.

This section introduces the generating abilities of ChatGPTs and C-dominant LLMs, the story continuation writing tasks for assessing their writing ability, and the NLP-based writing evaluation, and proposes the objectives and research questions at the end.

### LLMs’ generating ability

LLMs’ story-generating ability stems from their pretraining method and databases. LLMs were trained based on word embedding modeling, which grasps the semantic and syntactic elements in the sentence [6,7]. This modeling process enables them to predict the next word based on the fixed combination of tokens [8]. Consequently, LLMs are capable of generating and continuing what users prompt. Built upon extensive online datasets, encompassing materials from Wikipedia, news articles, books, websites, and social media [5,9], LLMs can grasp the patterns and connections inherent in language and hence generate responses for a wide array of language-related tasks, including text analysis, translation, and writing [9].

LLMs are designed with a hierarchical structure, attention mechanism, and prompt engineering techniques relating to language configuration and have the ability to process and generate texts with higher accuracy. The hierarchical structure captures different layers in input data and represents the relationships between sentences, paragraphs, and texts [7]. Meanwhile, the attention mechanism enables LLMs to focus on the most relevant portion of input data, thus leading to better comprehension of the context and improved performance in tasks such as translation and text generation [10]. Furthermore, the techniques, such as chain of thought (CoT), in-context learning (ICL), and reasoning and acting (ReAct), have improved LLMs’ reasoning ability and comprehension of context [11], rendering LLMs ability to generate more contextually appropriate texts. These mechanisms, along with pretraining, give LLMs formal linguistic competence, enabling them to understand vocabulary and form well-structured sentences [7].

### Generating ability of ChatGPTs and C-dominant LLMs

GPT-3.5 and GPT-4 exhibit fabulous abilities in text generation. GPT-3.5 responds to a variety of text-based requests in natural language [3,12], including English and Chinese. Pre-trained on various online sources, ChatGPT learns explicit language patterns and relationships, empowering itself with the capacity for text analysis, translation, and writing [9]. Additionally, it has been proven to be efficient in augmenting texts resembling authentic semantic scenarios [13], providing evidence that ChatGPT would be capable of extending given text in a reasonable manner. GPT-4 has been tested to significantly surpass GPT-3.5, offering enhanced world knowledge, problem-solving, reasoning skills [14], and gaining superior ability regarding text interaction and semantic comprehension [12]. Furthermore, GPT-4’s SAT Evidence-based Reading & Writing score of 710, compared to GPT-3.5’s 670 [14], further highlights its advanced text generation abilities.

ERNIE is capable of producing bilingual writing based on its unique training framework. ERNIE 3.0, a Chinese LLM, excels in bilingual writing due to its unique Continual Multi-Paradigms Unified Pre-training Framework, and can capture both common abstract features across tasks and task-specific top-level features [15]. This enhances ERNIE’s understanding of lexical, syntactic, and semantic information. Furthermore, ERNIE 3.0 is fine-tuned on both Chinese and English datasets and hence can efficiently handle tasks in the two languages [15].

SparkDesk (SPARK) is designed to possess the ability to generate both English and Chinese texts. However, no official introduction of SparkDesk has been available online to date, whereas previous research can support its ability to understand and generate both English and Chinese texts. For example, Zeng et al. [16] confirmed its Chinese text-generating ability by different Chinese exams. Also, the official website of SparkDesk claims its capability to complete English writing tasks. Furthermore, one focus of SparkDesk is on generating texts with abundant emotions and creativity, rendering the possibility that the text it produces may be full of imagination [17].

On this background, one shared feature of the above four LLMs is that they are competent at generating texts in both English and Chinese. Accordingly, this study is targeted at examining how the four models perform in actual text generation.

### Previous research on ChatGPTs and C-dominant LLMs’ abilities

Limited research has compared the abilities of different LLMs. Zeng et al. [16] evaluated 10 models, including GPT-4, SparkDesk, and ERNIE Bot, using Chinese academic exams, with GPT-4 performing best across six disciplines. Xu et al. [18] used the superCLUE benchmark (a comprehensive Chinese benchmark) and user ratings to assess these models’ problem-solving abilities in real-world scenarios, again showing GPT-4 outperforming others, while ERNIE Bot and SparkDesk ranked 4th and 5th. Despite these evaluations, no studies have yet assessed the models’ capacity for long-text generation.

A few studies have assessed LLMs’ ability to generate a text or story. Clark et al. [19] invited experts to evaluate the texts generated by humans and GPTs and found that GPTs underdid students in generating stories, news articles, and recipes. In contrast, Zhou et al. [5] showed that ChatGPT outperformed Chinese intermediate English learners in narrative writing, particularly in narrativity and word concreteness, with improved results from upgraded commands. Shin and Lee [20] noted that ChatGPT generated reading materials comparable to human experts in flow and expression. Callan and Foster [21] found LLMs capable of generating coherent paragraphs from one-sentence prompts. These studies converge to demonstrate that LLMs are able to generate stories or even continuations based on the existing plots.

To our knowledge, previous research has unanimously centered on ChatGPT’s writing ability [20,22,23], but no studies were found to compare the continuation writing tasks by ChatGPTs and C-dominant LLMs, hindering the audience from thoroughly understanding their respective superiorities and inferiorities in text generation.

### The story continuation task and assessment

To compare the LLMs’ writing abilities from different perspectives, we chose the story continuation writing task (SCWT) as our assessment task. The following is to introduce the SCWT and the evaluation indices adopted in this research.

The story continuation writing task requires multiple skills, including reading, comprehension, and writing. A SCWT demands writers to offer a reasonable ending for an incomplete story [24]. To this end, writers need to thoroughly understand the source story based on five dimensions, like “space, time, causality, intentionality, and protagonists” [25] before producing a natural upcoming event. On the basis of such a requirement, the SCWT is able to test writers’ ability to construct a cohesive text according to the given context. Furthermore, the SCWT has been confirmed to facilitate the transfer of language knowledge from reading to writing, supporting language learning by requiring writers to thoroughly engage with the source material and creatively mimic its style [26].

Accordingly, the SCWT can be deemed to be an appropriate task for assessing LLMs’ writing capacity. First of all, in order to provide a reasonable ending, the models should seamlessly continue a narrative, maintaining the tone, style, and direction of the original text. This assesses the models’ understanding of structure and narrative flow. Secondly, the characteristics of constructing an ending based on the given context of SCWT measure the models’ ability to track and reference prior plot over longer stretches of text. Thirdly, this research is dedicated to adopting both English and Chinese SCWT, providing an opportunity to evaluate the LLMs’ ability to adapt to different writing genres, tones, changes in plot, and language differences.

To better understand the differences among the four LLMs, this research aims to assess the cohesion, creativity, and formal linguistic competence manifested in the generated texts for the following reasons. Firstly, cohesion is particularly critical in SCWT, where writers must maintain coherence and discourse continuity with a given source text [24]. Secondly, creativity is an important feature of story continuation tasks, which require writers not only to align with the source text but also to generate novel, contextually appropriate developments, especially in familiar tasks [27].Thirdly, the formal linguistic competence reflecting LLMs’ underlying language proficiency (e.g., syntax and grammar) has been widely acknowledged [5,7,28]. Taken together, these three dimensions are complementary in assessing writing performance, consequently constituting LLMs’ SCWT ability from three aspects: textual alignment, narrative innovation, and linguistic form.

### Cohesion

Cohesion offers a valuable lens for evaluating continuation quality through linguistic features. It is a key criterion in assessing SCWT [24]. Defined as “relations of meaning that exist within the text” [29], cohesion is usually identified by explicit linguistic markers, such as connecting words, repetition, or references [30–32]. Cohesion contributes substantially to the semantic continuity and interaction between different parts of the text, making the understanding of logic and linguistic interaction possible [29] and enabling readers to connect ideas via lexical and grammatical cues [30]. Obviously, analyzing cohesive devices in SCWTs can reveal differences in writing abilities between ChatGPTs and C-dominant LLMs.

In general, writing cohesion is frequently assessed from three aspects, including referential cohesion, deep cohesion, and latent semantic analysis [33,34]. The first index, referential cohesion, refers to the repetition of characters or objects mentioned earlier [35] and is divided into local cohesion (word overlap at the sentence level) and global cohesion (word similarity across paragraphs) [36]. The referential cohesion in this research is assessed based on noun, argument, and stem overlap for English SCWT, while noun, word, and lexical word overlap for Chinese SCWT based on the tools leveraged. The second index, deep cohesion, is usually defined as the proportion of conjunctions used [37], particularly causal connectives, as they help readers follow the plot through cause-and-effect relationships [38,39]. In this research, we adopted connectives and conjunctions as indicators of deep cohesion.

### Creativity

Writing is a fertile soil for showing writers’ creativity. A well-recognized creative writing possesses an “authentic voice” for its readers to identify and pursue [40]. It usually expresses novel, surprising, valuable, and original ideas [41]. Furthermore, creativity and writing are closely interconnected as writing reflects writers’ creativity during the composing process and promotes the cultivation of creativity, while creativity facilitates the improvement of writing [42]. Being one crucial aspect of writing, creativity has been evaluated from different perspectives, such as creative fluency, flexibility, and originality based on rater judgements [42,43] and image, voice, and originality using computational tools [40].

However, the raters’ judgements are inevitably influenced by their subjective preferences due to inherent cognitive biases, individual aesthetic knowledge, and cultural backgrounds. Factors such as mood, experience, implicit expectations, and contextual framing also contribute to the variability in evaluations. Even with standardized rubrics, these human elements make thorough objectivity difficult to achieve. Therefore, this research decided to adopt the computational tools to objectively compare the writing creativity among LLMs.

Although creativity has been recognized as an inferiority of LLMs, some studies suggest they have the potential to generate creative responses. Boden [44] argued that AI techniques could generate creative ideas by combining familiar ideas, exploring unexpected conceptual spaces, and transforming previously impossible ideas into reality. Jennings [45] suggested that creative autonomy was achievable when integrated with a diverse community of creators and critics. Gómez-Camacho et al. [46] confirmed that ChatGPT could outperform students in imagination tests, generating high-quality, imaginative responses. These findings indicate that LLMs can produce creative writing, making creativity a key aspect of SCWT evaluation in the present study.

### Formal linguistic competence

The linguistic competence of LLMs includes both formal and functional aspects, with the former describing the ability to “produce and comprehend a given language” and the latter dealing with the capacity to generate texts with certain intentions [7]. The formal linguistic competence, focusing on the structural characteristics of sentences, i.e., phonology, morphology, lexical semantics, and syntax [7], has been believed to be a strength of LLMs [5,7,28]. The functional linguistic competence encompasses formal reasoning, world knowledge, situation model, and social reasoning, all of which have been regarded as weak parts of LLMs [7]. This research was centered on the formal linguistic competence to investigate the structural characteristics produced by four LLMs, so as to identify their diverse competences in constructing cohesion and creativity in the SCWT.

### NLP-based writing evaluation

For the purpose of objectively assessing the four LLMs’ writing cohesion and creativity, this study applied natural language processing (NLP)-based tools to encode the writing outputs. This section aims to provide information on the NLP tools used.

NLP tools are widely used to evaluate writing, with Coh-Metrix, CTAP, and LIWC being reliable in practice. Coh-Metrix, with over 100 indices, measures discourse characteristics such as referential cohesion, latent semantic analysis, and connectives, focusing on cohesion [47]. Gupta et al. [34] emphasized referential cohesion, measuring local and global cohesion through stem, noun, and argument overlap in narrative tasks. Wilson et al. (2017) adopted different indices to assess writing skills in terms of word, sentence, and discourse competence, including cohesion indices. Other studies further support Coh-Metrix’s effectiveness in measuring cohesion across different student groups [33,48].

CTAP (Common Text Analysis Platform) offers a usable program for Chinese text analysis. Since the Chinese Coh-Metrix is still underdeveloped and unavailable online, we turned to another computational tool specially designed for Chinese text analysis—the CTAP [49–51]. The CTAP is an open-source platform for linguistic complexity evaluation, including 196 indexes, much more competitive than other tools for Chinese text analysis. More importantly, this platform contains cohesive complexity indexes that are similar to those in Coh-Metrix, namely referential cohesion (local and global cohesion), as well as connectives.

LIWC is identified to be efficient for providing creativity indexes. Developed by Tausczik and Pennebaker [52], LIWC focuses on psychology-relevant categories in text based on explicit word usage. Kim et al. [53] demonstrated the reliability of KLIWC (Korean LIWC) in creativity research. Zedelius et al. [40] further validated LIWC for evaluating creative writing by capturing significant predictors related to image (i.e., insight, feel, body) and voice (i.e., authenticity, dictionary, all punctuation, comma, and informal language) [40], establishing useful evaluation rubrics for creativity in writing [54]. Based on Zedelius et al. [40], the present study incorporated the significant indices as the creativity variables.

### The present study

To summarize, previous research has gained four major achievements relating to the current topic. First, the four models have been announced to be capable of generating both English and Chinese texts, including continuation writing in a large sense. Second, the models have learned excellent linguistic competence. Third, the models have been confirmed to demonstrate creativity to some extent. Fourth, Coh-Metrix and LIWC have been proven to be valid in evaluating cohesion and creativity with respect to writing.

Despite the achievements, it remains unknown how the four models perform in the SCWT and whether they demonstrate ideal cohesion and creativity in text generation. In addition, the question remains open how these models perform in terms of formal linguistic competence in continuation writing.

Accordingly, the present study was conducted to compare the four AI models’ (i.e., GPT-3.5, GPT-4, ERNIE 3.0, and SparkDesk, the specific versions are presented in Table 1) performances in SCWT, from the perspectives of cohesion and creativity in particular. As a result, we intend to address the following questions.

**Table 1:** LLMs used in the present study.

LLMs	Version
GPT-3.5	gpt-3.5-turbo-0125
GPT-4	gpt-4-turbo-2024-04-09
ERNIE 3.0	ERNIE-Bot-turbo 10B version
SparkDesk	iFlytekSpark-13B

**RQ1:** How did the four LLMs perform in English and Chinese SWCTs from the perspective of cohesion and creativity?

**RQ2:** How were the cohesive variables in the produced English and Chinese continuations correlated with creativity?

**RQ3:** How did the four LLMs perform in English and Chinese SWCTs from the perspective of formal linguistic competence?

## Method

### Materials

This study adopted both English and Chinese SCWTs to assess the four models’ writing cohesion and creativity. Noteworthily, we specially chose writing tasks with no particular cultural specificity to eliminate the impact of culture on LLMs’ decisions and responses. The English SCWT was adapted from one of the 2023 Gaokao tests, telling a story about an encounter with a hummingbird in a cookout. The whole story contains 336 words and was rated 5.6 on the Flesch-Kincaid scale regarding readability, indicating it was easy to read. The four models were required to complete the task in about 150 words (as required in the Gaokao test). The instruction for the English SCWT includes, “阅读以下材料，根据其内容和所给段落续写一篇短文补充结局，字数为150左右” (Please read the following text and complement an English ending according to the given context with a word limit of around 150.).

The Chinese SCWT was adopted from Zhang [26], which depicts the Monkey mother asking her three monkey kids to live and work independently outside their familiar woods. The text includes 500 characters, specially tailored for CSL (Chinese as a Second Language) learners aged from 14–18, indicating similar reading difficulty to the English material. As commanded by Zhang [26], we asked the four models to produce texts in about 250 words: “阅读以下材料，根据其内容和所给段落续写一篇短文补充结局，字数为250左右” (Please read the following text and complement a Chinese ending according to the given context with a word limit of around 250.). The input texts of English and Chinese are provided in supporting information (S2 File).

### Measuring indices

To examine and compare the performances of the four LLMs in English and Chinese SCWTs, we coded and analyzed the overall performance, cohesion and creativity of the generated texts. For overall performance, we selected three metrics(narrativity, syntactic complexity, and word concreteness) for English SCWT, while four metrics (number of words, sentences, phrases, and characters) for Chinese SCWT. Altogether, these metrics were combined to constitute the criterion whereby a text’s complexity is judged and assessed. Cohesion, referential cohesion and deep cohesion were coded for SCWT in both languages, and additional latent semantic analysis were conducted for English SCWT. Creativity included image (insight, feel, and body) and sound (authenticity, informal language, dictionary, all punctuation, and comma)in this study. Supporting information (S1 File) presents all the metrics adopted in the current writing assessment.

As for the formal linguistic competence, given that no definite indices have been mentioned in previous research regarding LLMs, we chose to summarize and infer LLMs’ formal linguistic competence based on the results of overall performance, cohesion, and creativity. More specifically, three metrics involving grammar, syntax, word choice, and sentence length were adopted to measure the different formal linguistic competence displayed by the four LLMs.

### Procedure

**Fig 1:**
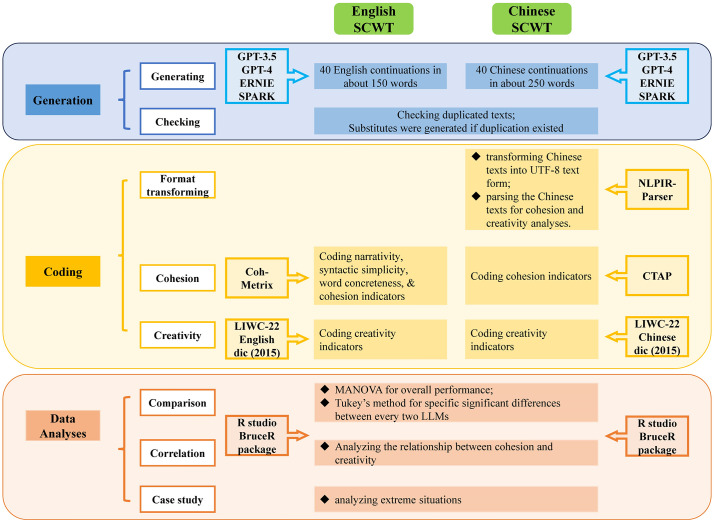
Research procedure.

The procedure adopted for this research was shown in Fig 1, with the NLP tools available as shown in Table 2. All the story generation was conducted in the end-user chat interface. Each continuation text was generated in individual chat box and each LLM was required to generate texts for 40 times, yielding 40 texts from each LLM as a result.

**Table 2:** NLP tools used in the present study.

NLP tools	Usage	Reliability metrics
NLPIR-Parser [55]	transforming text form acceptable for CTAP and CLIWC; parsing the Chinese texts for cohesion and creativity analyses.	Deterministic parsing; identical outputs across repeated runs
Coh-Metrix	calculating narrativity variables and the cohesive variables in English continuations.	Test–retest consistency (r = 1.00)
CTAP	calculating the cohesive variables in Chinese continuations.	Computational reproducibility (100%)
LIWC-22	calculating image and voice variables in English continuations through LIWC 2015 Dictionary (English).	Dictionary-based deterministic output
LIWC-22	calculating image and voice variables in Chinese continuations through LIWC 2015 Dictionary-Chinese (simplified) (V1.5).	Dictionary-based deterministic output

All the coding results were presented in S4 file.

Note. Reliability is defined in terms of computational stability and reproducibility rather than inter-rater agreement, as all tools are fully automated.

### Data analyses

After being decoded by the NLP tools, the statistical data representing the required variables were analyzed in R [56]. Specifically, the statistical data was analyzed using EMMEANS of the bruceR package [57] to conduct MANOVA (Multivariate Analysis of Variance) for overall comparison between ChatGPTs and C-dominant LLMs, and Tukey’s methods for post hoc pairwise comparisons, which include corrections for multiple comparisons to control the Type I error rate, for understanding specific significant differences between every two LLMs. Additionally, case studies were adopted to further analyze extreme situations, such as remarkably high or low scores in specific indices. Afterwards, correlation analyses were performed using the bruceR Corr tests [57] to investigate the relationship between cohesion and creativity.

## Results

### English continuation

#### Overall performance of LLMs’ English continuation.

Based on the statistical data by LIWC-22, we first summarized the total word count (WC), words per sentence (WPS), and big words (BW, words longer than 7 letters) that appeared in the English continuations by the four LLMs. The results showed that GPTs generated an average of 278.9 words, 20.8 words per sentence, and 22.36 big words, indicating the highest fluency [46], whereas GPT-4 produced an average of 189.85 WC, 20.64 WPS, and 25.78 BW. Among C-dominant LLMs, ERNIE generated an average of 223.1, 19.58, and 20.39 words while SPARK generated 185.05, 19.55, and 20.71 words, respectively.

Table 3 shows the percentiles, namely means and SDs of continuation samples generated by the four LLMs in the three indices, containing narrativity, syntactic simplicity, and word correctness. A MANOVA was conducted to examine whether a significant difference exists among the four LLMs. The results indicated an overall significant difference of Group (*F*(3, 156) = 28.172, *p* < .001), and hence follow-up three one-way ANOVAs were conducted, revealing striking differences in narrativity, syntactic simplicity, and word correctness.

**Table 3:** Overall performance of the four LLMs.

variables	GPT-3.5		GPT-4		ERNIE		Spark		*F*(3,156)	*p*	*η²p*
	*M*	*SD*	*M*	*SD*	*M*	*SD*	*M*	*SD*			
Narrativity	67.41	11.67	29.98	14.99	82.28	11.45	81.25	12.44	43.158	<.001***	0.454
Syntactic simplicity	22.32	12.72	22.38	13	23.87	14.79	25.96	15.83	17.527	<.001***	0.252
Word concreteness	75.94	12.55	79.89	16.05	62.15	24.35	76.45	16.47	26.474	<.001***	0.337

**Fig 2:**
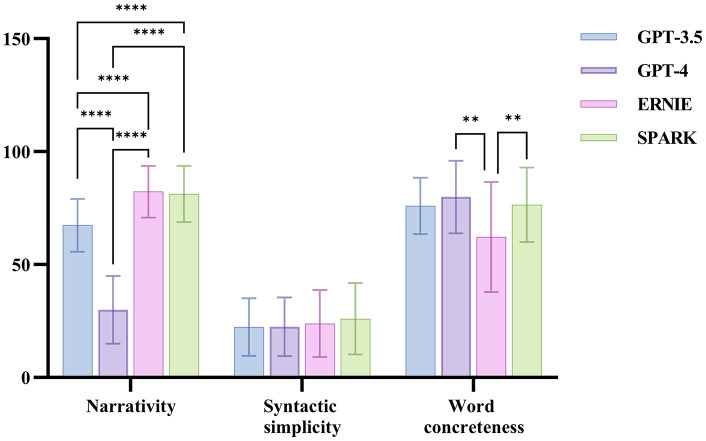
Multiple comparisons on three indices of continuation writing by the four LLMs.

Fig 2 presents the results of multiple comparisons using Tukey’s method, indicating that in “narrativity”, GPT-3.5 outdid GPT-4 but lagged behind ERNIE and SPARK, and simultaneously GPT-4 underperformed ERNIE and SPARK (Note that all the results for comparison are specified in supporting information (S3 File)). With respect to “word correctness”, GPT-3.5 and GPT-4 were found to perform better than ERNIE, while ERNIE fell behind SPARK. As for “syntactic simplicity”, no significant difference was found between any two LLMs.

The results indicated the extremely low score by GPT-4 in narrativity. A case study further digging into this phenomenon found dense abstract, argumentative, and philosophical expressions existing in GPT-4 generated texts, as shown in the underlined parts in Exs.1 and 2. These expressions, though beautiful, lacked descriptions of concrete actions and plots.

*Ex.1: The hummingbird, in its fragile grace, seemed to carry a message – a message of gratitude and the invisible threads that connect all living beings. It was a simple yet powerful affirmation of life’s beauty, witnessed in the midst of an ordinary day, turning it into an unforgettable moment of connection and wonder.*

*Ex.2: Life’s busy rhythm often makes us overlook the subtle wonders of nature. But experiences like these are reminders of the astonishing connections we share with the world around us. The hummingbird became a symbol of hope and gratitude, a gentle reminder that beauty and kindness exist in the smallest and most unexpected places. Its spirit, in its miraculous minuteness, brought a magical perspective to ordinary days, turning simple moments into everlasting memories.*

In addition, more emphases were put on the inner feelings instead of the events to drive the plot forward. In other words, GPT-4 leveraged more introspective phrases: *lingered in my mind, my thoughts, I felt.* The bird here appeared only as part of the description, with very few interactions between the person and the bird. For example, in Ex.3, rather than depicting external actions or interactions, GPT-4 described the main character’s recalling, which is introspective writing instead of narration. Similarly, the Ex.4 demonstrated that GPT-4 delineated lines to illustrate another encounter without elaborate plotlines of their interactions but with static descriptions of the character’s feelings.

*Ex.3: As I drove back home, the hummingbird’s bright eyes and miraculous return lingered in my mind, painting the skies of my thoughts with strokes of wonder and warmth. It seemed like a simple act of kindness had woven an invisible thread connecting our souls, a thread that pulsed with life’s beautiful mysteries.*

*Ex.4: One peaceful morning, as I was savoring my coffee in the garden, a familiar flutter caught my attention. To my astonishment, a hummingbird appeared, hovering near a flower before darting closer to me. Our eyes met, sharing an unspoken bond, and I felt an overwhelming sense of gratitude.*

Moreover, ERNIE demonstrated a markedly lower score in word concreteness. Our analysis of the texts by ERNIE showcased its excessive use of abstract words, such as vocabulary for reflection and value, as indicated in Examples 5 and 6. The three paragraphs contained a noticeable density of abstract terms, including nouns, verbs, and adjectives, which do not portray any concrete objects, actions, events, or characteristics as illustrated in Table 4.

**Table 4:** Sample abstract words used by ERNIE.

Example	Types	words
Ex.5	Noun	appreciation, beauty, fragility, respect, action, impact, web of life, survival
	Verb	taught, take, vowed, protecting, preserving
	Adjective	deep, profound, vital, newfound
Ex.6	Noun	rescue, thankfulness, resilience, interconnectivity, beliefs, circumstances, courage
	Verb	reminded, become trapped, seek
	Adjective	apparent, limiting, toughest

*Ex.5: The experience left me with a deep appreciation for the beauty and fragility of life, and a newfound respect for the smallest of creatures. It taught me that we are all interconnected, and that every action we take, no matter how small, can have a profound impact on the world around us.*

*As I drove away from the farm that day, I looked out at the passing scenery with newfound eyes. I saw the fields and trees and flowers, not just as beautiful landscapes, but as vital parts of the web of life. And I vowed to do my part in protecting and preserving that web, not just for myself but for all the little hummingbirds and other creatures who relied on it for their survival.*

*Ex.6: The hummingbird’s rescue and apparent thankfulness for it spoke to me about the resilience of life and the interconnectivity of everything. The spider-webs that had trapped her were a reminder of how we can become trapped by our own limiting beliefs or circumstances. Yet, even when life seems toughest, there is always a way to break free, if only we have the courage to seek it.*

In summary, the results showed that GPT-4 performed worse than the other three LLMs in narrativity, whereas SPARK scored the lowest in word concreteness.

#### Cohesion in English continuation.

Table 5 demonstrates the means and SDs of cohesive indices retrieved from English continuation by the four LLMs. A MANOVA on the overall cohesion performance indicated significant differences among the four LLMs (*F*(3,156) = 11.763, *p* < .001). Additionally, one-way ANOVA tests revealed significant differences in local noun overlap (LNO), local argument overlap (LAO), connectives, causal connectives, global noun overlap (GNO), global argument overlap (GAO), global stem overlap (GSO), LSA sentence, and LSA all sentence as well. By contrast, no major significance was recognized in local stem overlap.

**Table 5:** Cohesion of the four LLMs in English continuation.

variables	GPT-3.5		GPT-4		ERNIE		SPARK		*F*(3,156)	*p*	*η²p*
	*M*	*SD*	*M*	*SD*	*M*	*SD*	*M*	*SD*			
Referential cohesion (Local)											
LNO	0.11	0.09	0.16	0.17	0.13	0.14	0.06	0.07	4.93	.003**	0.087
LAO	0.45	0.18	0.36	0.18	0.65	0.18	0.60	0.17	23.764	<.001***	0.314
LSO	0.19	0.13	0.23	0.20	0.22	0.16	0.16	0.13	1.716	0.166	0.032
Referential cohesion (Global)											
GNO	0.10	0.01	0.08	0.01	0.09	0.02	0.07	0.01	26.474	<.001***	0.337
GAO	0.50	0.02	0.46	0.03	0.51	0.03	0.51	0.03	27.284	<.001***	0.344
GSO	0.13	0.02	0.11	0.02	0.12	0.02	0.10	0.01	28.53	<.001***	0.354
Deep cohesion											
Connectives	88.38	13.12	69.98	15.64	94.56	18.91	97.07	16.90	22.599	<.001***	0.303
Ca-Connectives	14.60	7.42	9.08	6.11	19.13	10.07	23.39	9.76	20.87	<.001***	0.286
Latent semantic analysis											
LSA-Sen	0.16	0.01	0.16	0.01	0.15	0.01	0.16	0.01	6.214	<.001***	0.107
LSA-All	0.11	0.01	0.10	0.01	0.10	0.01	0.11	0.01	8.595	<.001***	0.142

*Note*. LNO: Local noun overlap; LAO: Local argument overlap; LSO: Local stem overlap; GNO: Global noun overlap; GAO: Global argument overlap; GSO: Global stem overlap; Ca-Connectives: Causal connectives; LSA-Sen: LSA adjacent sentences; LSA-All: LSA all sentences.

**Fig 3:**
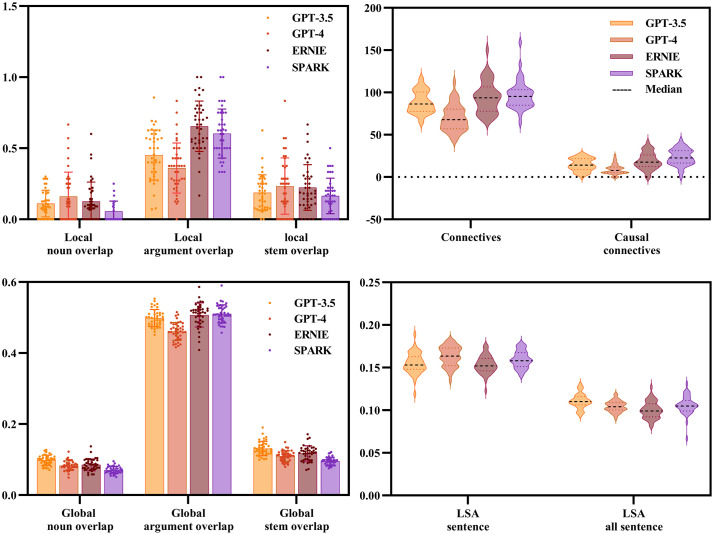
Multiple comparisons on cohesion in English continuation.

Multiple comparisons utilizing the Turkey method showed (in Fig 3) that SPARK gained lower scores in “LNO” than GPT-3.5, GPT-4, and ERNIE, whereas no significance was observed among the other three LLMs. In “LAO”, GPT-3.5 and GPT-4 performed worse than ERNIE and SPARK. As for “connectives”, GPT-3.5 performed better than GPT-4, but worse than SPARK, unexpectedly. Similar inferiority was observed in GPT-4 relative to ERNIE and SPARK.

In “causal connectives”, GPT-4 showed poorer performance than ERNIE and SPARK. Additionally, GPT-3.5 fell behind SPARK.

In terms of “GNO”, GPT-3.5 gained higher scores than GPT-4, ERNIE, and SPARK. GPT-4 showed better power than SPARK. In addition, SPARK fell short of ERNIE’s performance. Concerning “GAO”, GPT-4 obtained the worst performance as compared to GPT-3.5, ERNIE, and SPARK. In “GSO”, GPT-3.5 outperformed the other three LLMs, ranking the highest and followed by ERNIE, which slightly won SPARK.

For the aspects of “LSA”, GPT-4 outperformed GPT-3.5 and ERNIE in “LSA adjacent sentences” (LSA sentence). Furthermore, GPT-3.5 outdid GPT-4 and ERNIE in “LSA all sentences” in the paragraph.

In addition, the results indicated a notably low score by SPARK in LNO. A further exploration of the texts demonstrated very few repetitions of semantically similar nouns in adjacent sentences by SPARK, as shown in Ex. 7 and 8. Across the three neighboring sentences in Ex.7, there is an absence of noun repetitions, either literal or semantic (nouns in 1st sentence: experience, hummingbird, kitten, lesson, compassion, empathy; in 2nd sentence: being, feelings, respect, care; in 3rd sentence: day, mission, creatures, act, kindness, world, difference). A similar situation also occurs in Ex.8.

Ex.7: *The experience with the hummingbird and the kitten taught me a valuable lesson about compassion and empathy. It showed me that every living being has feelings and deserves our respect and care. From that day on, I made it my mission to always be kind and compassionate towards all creatures great and small, knowing that even the smallest act of kindness can make a world of difference.*

*Ex.8: As I drove away from the farm, I felt a sense of peace and contentment wash over me. I knew that I would never forget the hummingbird who had taught me such an important lesson. And as I continued on my journey, I made a promise to myself to always be kind and compassionate towards all living creatures, no matter how small or insignificant they may seem.*

Furthermore, GPT-4 received remarkably low scores in terms of LAO, Connectives, and Ca-connectives. For LAO, the generated texts demonstrated very few repetitions of augments, i.e., subject and object, in adjacent sentences, as illustrated in Ex. 9, where the subjects in the three sentences were *We, The air,* and *The little hummingbird*, without any semantic or structural overlap. As for connectives, Ex. 10 and 11 suggested that a minimal number of conjunctions were adopted to connect sentences. For instance, the sentences in Ex.10 were tightly connected through logical progression and cause-effect relationships. However, they lacked any explicit connectives such as therefore, thus, consequently, because, as, since, moreover, furthermore, or then. Similarly, although time sequence was contained in the three sentences in Ex. 11, no temporal conjunctions, such as when and after, were used. Finally, rare instances of causal conjunctions led to GPT-4’s lowest score in Ca-connectives. As indicated in Ex.12, despite the apparent causal relationship between the three sentences, no explicit conjunctions were adopted.

*Ex.9: We all stood there, stunned and touched by the unexpected visit. The air was filled with a soft, magical aura, making the moment feel surreal. The little hummingbird, with her vibrant feathers, had brought a miraculous touch to our simple cookout, turning it into a moment of awe and wonder.*

*Ex.10: The drive back home was filled with thoughts of the extraordinary encounter. The hummingbird’s bright eyes and delicate grace were etched in my memory, creating a sense of wonder and warmth. It felt as though nature had allowed me a rare glimpse into its beautiful mysteries, offering a moment of connection that was profoundly moving.*

*Ex. 11: One summer, I received an invitation for another cookout at the same farm. Excited, I arrived early. The farm had changed over the years – the old milking house had been restored, and in place of the broken window was a beautiful stained glass depicting a hummingbird.*

*Ex.12: Driving back, the image of the hummingbird’s bright eyes filled my thoughts. It was a silent communication, a soft thank-you from one life to another. The universe seemed to echo with a subtle, yet powerful reminder of the beauty and vulnerability that life encapsulates.*

In conclusion, GPT-4 fell short of the three LLMs regarding the three indexes, while ERNIE and SPARK performed much better.

#### Creativity in English continuation.

**Fig 4:**
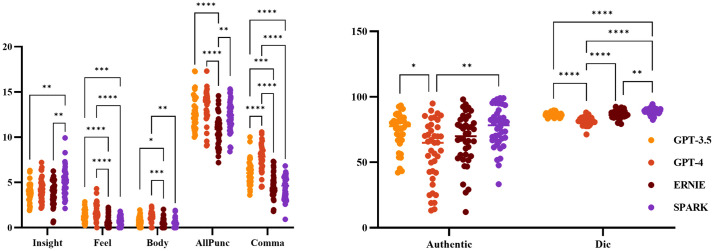
Multiple comparisons of creativity in English continuation.

Table 6 presents the descriptive results and comparison results concerning creativity from English continuation generated by the four LLMs. To gain insight into creativity, MANOVAs were conducted, revealing significance in insight, feel, body, all punctuation, comma, authenticity, and dictionary. The informal language index was removed because only 15 out of 160 continuations had successfully extracted values. Fig 4 shows the results of the comparison in English creativity.

**Table 6:** The creativity of the four LLMs in English continuation.

variables	GPT-3.5		GPT-4.0		ERNIE		SPARK		*F*(3, 156)	*p*	*η²p*
	M	SD	M	SD	M	SD	M	SD			
insight	3.977	0.977	4.306	1.222	3.924	1.299	5.073	1.534	6.916	<.001 ***	0.117
feel	1.363	0.591	1.651	0.909	0.648	0.554	0.745	0.521	21.377	<.001 ***	0.291
body	0.747	0.494	1.040	0.567	0.410	0.495	0.503	0.572	11.189	<.001 ***	0.177
AllPunc	12.872	1.824	13.361	1.758	10.768	1.728	12.349	1.699	16.488	<.001 ***	0.241
Comma	6.114	1.358	7.661	1.504	4.649	1.340	4.438	1.289	47.333	<.001 ***	0.477
Authentic	74.324	13.631	58.479	23.290	67.431	19.781	76.558	15.563	7.773	<.001 ***	0.130
Dic	86.734	1.616	81.648	3.124	86.730	3.014	89.108	2.444	57.678	<.001 ***	0.526
image	−1.867	1.406	−1.615	1.549	−2.866	1.333	−3.825	1.910	16.684	<.001***	0.243
voice	6.576	14.290	−2.147	22.770	−3.881	19.441	4.237	14.609	3.05	.030*	0.055

Note. AllPunc: all punctuation; Authentic: authenticity; Dic: dictionary words.

Specifically, in the index “insight”, SPARK outdid GPT-3.5, GPT-4, and ERNIE, whereas no significant effect was found among GPT-3.5, GPT-4, and ERNIE. In the index “feel”, GPT-4 gained significantly higher scores than ERNIE and SPARK. Similarly, GPT-3.5 outperformed ERNIE and SPARK, while no significant difference was found between GPT-3.5 and GPT-4, SPARK and ERNIE. About the index “body”, GPT-4 manifested the greatest performance, outdoing ERNIE and SPARK. Furthermore, GPT-3.5 surpassed ERNIE in this regard.

On the index “all punctuation”, ERNIE gained poorer performance than GPT-3.5, GPT-4, and SPARK. And for “Comma”, GPT-4 exhibited better performance than GPT-3.5, ERNIE, and SPARK. Additionally, GPT-3.5 also outperformed the C-dominant LLMs. As for “authenticity”, GPT-4.0 lagged behind GPT-3.5 and SPARK. As for the “dictionary”, GPT-4 also lagged behind the other three LLMs. SPARK outdid GPT-3.5 and ERNIE.

Notably, the results presented two extremes of performance between GPTs and C-dominant LLMs in feel and body. A closer look at the texts revealed that compared with GPTs, C-dominant LLMs utilized significantly fewer expressions indicating feeling and body descriptions. For example, GPT-4 adopted dense feeling-related words (e.g., heartwarming, felt, feel a mix of emotions, memorable in Ex. 14) and body-relevant expressions (e.g., bright eyes, soft humming, tiny wings, and close to my face in Ex. 15) in adjacent sentences. By contrast, ERNIE hardly described the hummingbird’s appearance or adopted body-related words as shown in Ex.16, in which although the hummingbird was mentioned six times in one text, no bodily description was elaborated. A similar situation can be found in the index “feel” that ERNIE lacked descriptive details regarding feelings, as shown in Ex.17 where the expressions for feeling were scarce and distributed in different texts, different from the dense use in the same text.

Ex.14: *All of us stood there, engulfed in a brief silence, absorbing the unusual yet heartwarming encounter. It felt as if nature itself had paused to allow us to experience this extraordinary connection. I couldn’t help but feel a mix of emotions, as the hummingbird had chosen to say her goodbye, making the moment remarkably special and memorable.*

Ex.15: *The bird’s bright eyes and soft humming filled my thoughts. The connection we shared was mystical, as though there was a silent communication between our souls. Her tiny wings, that once struggled in desperation, had fluttered so close to my face, breathing gratitude and a unique bond that left me mesmerized.*

*Ex.16: I visited a local animal rescue center to learn more about hummingbirds.*

*The staff was extremely knowledgeable and eager to share information about the different species of hummingbirds found in the area.*

*One of the suggestions was to create a hummingbird sanctuary in my own backyard.*

*It could include native plants and flowers that attract hummingbirds, such as salvia, echinacea, and penstemon.*

*In addition to attracting hummingbirds, my yard could provide a safe haven for other wildlife species. I could install a birdhouse or two for bluebirds or chickadees,*

*By creating a haven in my yard for hummingbirds and other wildlife, I will be providing a safe space for them to thrive and multiplying my goodwill towards nature.*

*Ex.17: The encounter left me feeling humbled and connected to the natural world. It reminded me that we are all part of a larger, interconnected ecosystem and that every living thing has value and a right to exist. (round 14)*

*The encounter with the hummingbird had left me feeling as though we had shared a special connection. (round 19)*

*It was a unique experience that left me feeling grateful and appreciative for the beauty and connection that nature brings into our lives. (round 27)*

For the overall performance in “image”, GPT-3.5 and GPT-4 outdid ERNIE and SPARK. Nevertheless, the differences were not significant in overall writing “voice” between every two LLMs, despite the significance across four LLMs.

### Chinese continuation writing

#### Overall performance by the four LLMs.

In the Chinese writing part, 200 valid texts were collected for analysis. After comparing the linguistic features provided by CTAP and LIWC and the indexes for Chinese complexity proposed by Liu et al. [58], we adopted some of the indexes in Liu et al. [58] to calculate text features, including total words, total phrases, total sentences, number of high-stroke-count (HSC), intermediate-stroke-count (ISC), and low-stroke-count (LSC) characters, and number of two-character and three-character words. Table 7 shows the descriptive results based on the features analyzed. The results revealed that ERNIE and SPARK produced more complex sentences, with more total words, three-character and two-character phrases, and ISC.

**Table 7:** Average text feature count in English and Chinese continuations.

LLMs	total words	sentences	phrases	three-character phrase	two-character phrase	HSC	ISC	LSC
GPT-3.5	509.53	37.23	324.75	5.30	133.93	1.83	142.20	365.35
GPT-4	438.08	31.68	287.40	5.30	133.93	1.58	122.43	323.96
ERNIE	517.08	37.03	330.90	6.08	165.48	1.35	146.08	369.70
SPARK	504.10	36.57	323.08	6.35	156.68	0.83	143.95	359.40

#### Cohesion of Chinese texts by the four LLMs.

**Fig 5:**
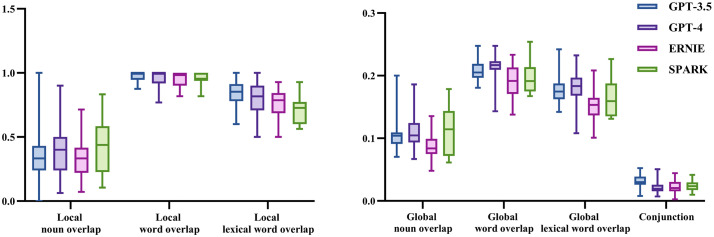
Multiple comparisons on cohesion in Chinese continuation.

Table 8 provides means and SDs of local and global indexes of cohesion in the Chinese continuation outputs by the four LLMs. A MANOVA was initially adopted to examine the overall difference in cohesion, displaying significance among the four LLMs (*F*(3,156) = 7.826, *p* < .001). The follow-up one-way ANOVA tests were employed to further analyze cohesion indexes, finding significance in local noun overlap, local word overlap, local lexical overlap, conjunction, global noun overlap, global word overlap, and global lexical overlap. Fig 5 displays the results of the comparison in Chinese cohesion.

**Table 8:** Cohesion of the Four LLMs in Chinese continuation.

variable	GPT-3.5		GPT-4		ERNIE		SPARK		*F*(3,156)	*p*	*η²p*
	*M*	*SD*	*M*	*SD*	*M*	*SD*	*M*	*SD*			
Referential cohesion (local)											
LNO	0.350	0.170	0.330	0.450	0.330	0.140	0.430	0.200	2.350	0.075	0.043
LWO	0.980	0.040	0.950	0.060	0.950	0.060	0.960	0.050	2.799	0.042*	0.051
LLWO	0.840	0.100	0.800	0.110	0.760	0.110	0.710	0.100	11.977	<.001***	0.187
Referential cohesion (global)											
GNO	0.100	0.020	0.110	0.020	0.090	0.020	0.110	0.040	6.616	<.001***	0.113
GWO	0.210	0.020	0.210	0.020	0.190	0.030	0.200	0.020	10.441	<.001***	0.167
GLWO	0.180	0.020	0.210	0.020	0.150	0.020	0.160	0.030	11.383	<.001***	0.180
Deep cohesion											
conjunction	0.030	0.010	0.020	0.010	0.020	0.010	0.020	0.010	11.342	<.001***	0.179

Note. LNO: local noun overlap; LWO: local word overlap; LLWO: local lexical word overlap; GNO: global noun overlap; GWO: global word overlap; GLWO: global lexical word overlap.

Follow-up multiple comparisons using Tukey’s method disclosed detailed differences among the LLMs. No significant difference was observed in “local noun overlap”. While the four LLMs displayed no significant discrepancy with each other in “local word overlap”, they showed differences in “local lexical overlap”. Specifically, GPT-3.5 gained higher scores than ERNIE and SPARK, and GPT-4 outperformed SPARK. In “conjunction”, GPT-3.5 manifested its best power, upstaging GPT-4, ERNINE, and SPARK.

In “global noun overlap”, ERNIE showed a lack of ability in comparison with GPT-3.5, GPT-4, and SPARK. For “global word overlap”, two GPTs presented a dominant advance compared with the two C-dominant LLMs. As for “global lexical overlap”, GPT-4 surpassed ERNIE and SPARK, whereas GPT-3.5 scored higher than ERNIE.

The results implied the noticeably low score by SPARK in LLWO, suggesting a lack of lexical word overlap produced in adjacent sentences. For example, Ex.17 described the jobs of the three monkeys without very few overlaps of key lexical words. Instead, SPARK advanced the plot by semantically different verbs and noun phrases, namely verbs like 找到-工作-省吃俭用-储蓄-找到-喜欢-通过-得到-成为-拥有-被签下 (find (a job) – work – live frugally – save – find (something you love) – persist – gain – become – own – get signed) and nouns like餐馆—工作—钱—科技公司—程序员—工作—问题—努力—智慧—认可—尊重—模特—外貌—个性—模特公司—合同 (restaurant – job – money – tech company – programmer – job – problems – effort – intelligence – recognition – respect – model – appearance – personality – modeling agency – contract).

By contrast, GPT-3.5 utilized semantically similar or consistent verbs and noun phrases to describe their jobs as shown in Ex.18, namely verbs like结交-合作经营-过上-找到-结交-找到-成为-吸引-取得-积极参与-用-帮助 (make connections – collaborate – build a better life – discover – make connections – discover – become – attract – achieve – actively participate – apply – help) and nouns like诚实和正直-忠诚的朋友-小店铺-幸福的生活-聪明伶俐的头脑-高薪的工作-专业人士-聪明才智-问题-聪明的朋友-机会-受欢迎的演员-美貌和可爱-粉丝-成功-慈善事业-需要帮助的人 (honesty and integrity – loyal friends – a small shop – a happy life – smartness – a high-paying job – a professional – smart– challenges – smart friends – opportunities – a popular actor – beauty and charm – fans – success – charity work – people in need)

*Ex. 17: 猴听话在一家餐馆找到了一份端盘子的工作，每天辛勤地工作着，用自己省吃俭用的钱储蓄起来。猴机灵则在一家科技公司找到了一份程序员的工作，他喜欢思考问题，通过自己的努力和智慧，他逐渐得到了同事们的认可和尊重。而猴美丽则成为了一名模特，她拥有迷人的外貌和自信的个性，很快就被一家知名模特公司签下了合同。（The Monkey Tinghua found a job as a waiter in a restaurant, working diligently every day and saving money through frugality. The Monkey Jiling landed a job as a programmer at a tech company. He enjoyed solving problems, and through his hard work and intelligence, he gradually earned the recognition and respect of his colleagues. Meanwhile, the Monkey Meili became a model. With her charming appearance and confident personality, she was quickly signed by a well-known modeling agency.）*

*Ex. 18: 猴听话因为他的诚实和正直，很快就结交了一群忠诚的朋友，大家一起合作经营了一家小店铺，生意兴隆，过上了幸福的生活。猴机灵则运用他聪明伶俐的头脑，找到了一份高薪的工作，成为一位受人尊敬的专业人士。他利用聪明才智，解决了许多问题，也结交了许多聪明的朋友，生活 充实而快乐。而猴美丽也在外面的世界里找到了自己的机会，她成为一名受欢迎的演员，因为她的美貌和可爱，吸引了众多粉丝。她不仅在表演事业上取得了成功，还积极参与慈善事业，用她的知名度来帮助需要帮助的人。(Because of his honesty and integrity, the Monkey Tinghua soon made a group of loyal friends. Together, they ran a small shop, and business flourished. They lived a happy and fulfilling life. The Monkey Jiling, using his quick wit and sharp mind, found a high-paying job and became a respected professional. He solved many problems with his intelligence and made many smart friends, leading a rich and joyful life. As for the Monkey Meili, she also found her opportunity in the outside world—she became a popular actress. Her beauty and charm attracted many fans. Not only did she achieve great success in her acting career, but she also actively participated in charity work, using her fame to help those in need.)*

In summary, the results indicate that GPT-4 showed obviously a better performance than GPT-3.5, ERNIE, and SPARK in global cohesion. Meanwhile, GPT-3.5 presented the highest general performance, followed by the two C-dominant LLMs, with GPT-4 as the worst.

#### Creativity of Chinese texts by the four LLMs.

Table 9 displays the descriptive and MANOVA results of creativity variables in Chinese continuations by four LLMs. The MANOVA results showed significant differences among the four LLMs in insight, all punctuation, informal language, and dictionary. The comma index was deleted because LIWC-22 extracted no value for all LLMs, and the authenticity index was not involved because this index was not contained in the coding results exported by LIWC-22.

**Table 9:** The creativity of the four LLMs in Chinese continuation.

variables	GPT-3.5		GPT-4.0		ERNIE		SPARK		*F* (3, 156)	*p*	*η²p*
	M	SD	M	SD	M	SD	M	SD			
insight	9.493	1.601	8.727	1.760	8.549	1.606	8.766	1.391	2.735	.046 *	0.050
feel	0.132	0.216	0.232	0.281	0.142	0.218	0.168	0.244	1.402	0.244	0.026
body	0.163	0.214	0.224	0.348	0.211	0.318	0.077	0.137	2.475	0.064	0.045
AllPunc	0.000	0.000	0.383	0.715	0.178	0.305	0.054	0.255	6.897	<.001***	0.117
informal	6.616	1.069	6.443	1.369	6.384	1.051	7.540	1.346	7.824	<.001***	0.131
Dic	85.782	2.423	84.228	3.263	85.216	2.622	86.560	3.357	4.445	.005 **	0.079
image	−9.198	1.746	−8.271	1.973	−8.196	1.704	−8.521	1.415	2.811	.041*	0.051
voice	−79.166	2.716	−77.403	3.765	−78.654	2.716	−78.966	2.669	2.755	.044*	0.050

**Fig 6:**
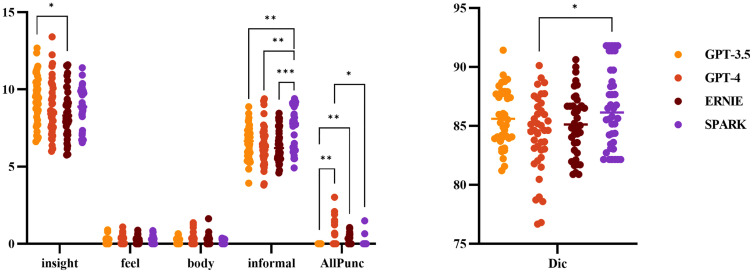
Multiple comparisons of creativity in Chinese continuation.

Multiple comparisons using the Turkey method demonstrated (Fig 6) that no significant difference was identified among the four LLMs in sight, body, and feel. As for “dictionary”, SPARK gained higher than GPT-4, while no significant discrepancy was observed among GPT-3.5, GPT-4, and ERNIE.

Regarding “informal language”, SPARK exhibited the best ability, outperforming GPT-3.5, GPT-4, and ERNIE. In the aspect of “all punctuation”, GPT-4 presented to obtain the highest score, outdoing GPT-3.5 and SPARK, whereas no significant difference was found between GPT-4 and ERNIE.

The results also manifested SPARK’s lowest performance in terms of informality, mainly resulting from the dense use of oral expressions. For instance, in Ex.19, SPARK generated phrases, such as “摆摊”, “回头客”, “生意红火”, and “赚了些钱” (“setting up a street stall,” “returning customers,” “business is booming,” and “made some money”), which are all typical examples of informal and colloquial expressions used to describe small-scale economic activity, instead of formal economic terminologies, such as “经营摊位”, “建立客户忠诚度”, “客流量增长”, and “盈利” (“operating a retail booth,” “building customer loyalty,” “increased foot traffic,” or “generating profit”). Similarly, Ex. 20 contained phrases “起初”, “渐渐地”, and “最后” (“Initially,” “gradually,” and “finally”), which were vague temporal markers. Further, the expressions like “不断地”, “很多”, and “一大笔” (“constantly,” “a lot,” and “a large sum”) were colloquial and somewhat exaggerated, which is evidently different from formal texts characterized by the use of more precise and cautious quantitative descriptions.

Ex.19: *猴听话在城市的街头摆了一个小摊，卖着自己亲手做的水果沙拉。他诚实守信，生意很快就红火起来。许多顾客都被他的真诚和勤奋所打动，纷纷成为他的回头客。猴听话虽然赚了一些钱，但他并没有贪婪，而是把大部分的钱都寄回了家，让猴妈妈和兄弟姐妹们过得更好。*

Ex. 20: *起初，生意并不是很好，但是他没有放弃。他不断地学习新的知识，努力提高自己的经营能力。渐渐地，他的店铺开始吸引了很多顾客，生意越来越好。最后，他成为了一名成功的商人，拥有了自己的公司和一大笔财富。*

Moreover, among the four LLMs, GPT-3.5 gained a notably lower score in image, probably manifested by the unspecific details. For instance, when required to describe their initial experience and first impression on moving to a new place, GPT-3.5 and ERNIE conveyed different degrees of detail. More specifically, Ex.21 incorporated abstract concepts, such as “外面的世界” and “不同于森林的世界” (“outside world” and “this world so different from the forest”), lacking any substantive description that concretizes their characteristics. Likewise, the concepts of “陌生”和“适应” (“unfamiliar” and “adapted”) were not supplemented with specific actions or representations, such as a detailed description of the “outside world” and the experience of “adaptation”. As a direct result, the absence of multisensory details (visual, auditory, or imagery) results in a lack of vividness and sensory engagement in the depiction. By contrast, Ex.22 contained details regarding the city, such as “宽阔的道路，高大的楼房，喧嚣的城市” (“wide roads”, “tall buildings”, “bustling cities”) and a vivid description of their reactions with actions and psychological adjectives, i.e., “一边走，一边好奇地观察着周围的一切。” (As they walked, they curiously observed everything around them).

*Ex.21:猴听话、猴机灵和猴美丽离开了自己的家，走进了外面的世界。一开始，他们对新的环境感到陌生，但随着时间的推移，他们渐渐适应了这个不同于森林的世界 (The Monkey Tinghua, the Monkey Jiling, and the Monkey Meili left their home and stepped into the outside world. At first, they felt unfamiliar with the new environment, but as time went by, they gradually adapted to this world so different from the forest.)。（GPT-3.5）*

*Ex.22: 经过好几天的行程，他们终于走出了森林，来到了他们从未见过的世界。这里有着宽阔的道路，高大的楼房，喧嚣的城市和各种各样奇怪的东西。他们一边走，一边好奇地观察着周围的一切 (After several days of travel, they finally emerged from the forest and arrived in a world they had never seen before. There were wide roads, tall buildings, bustling cities, and all sorts of strange and unfamiliar things. As they walked, they curiously observed everything around them.)。(ERNIE)*

Overall, although significant differences were recognized in image across the four LLMs, no significant discrepancy was found between every two LLMs, and the situation was so in voice.

### Correlations between cohesion and creativity

**Fig 7:**
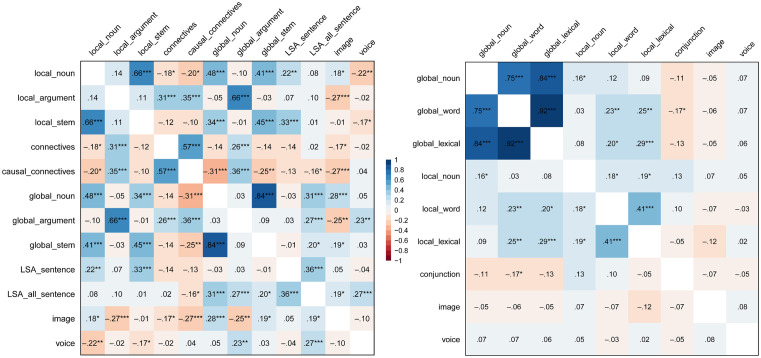
Correlations between cohesion and creativity of English (left) and Chinese (right) continuations.

To further support the interpretation of the observed differences in cohesion and creativity across models, we conducted correlation analyses between selected cohesive and creative indices (see Fig 7). This analysis was intended as a complementary examination to the internal relationships between textual organization and creative expression, rather than as a test of strong causal hypotheses. For English continuation, image was positively related to local noun (*r* = 0.18, *p* = .025), global noun (*r* = 0.28, *p* < .001), and LSA all sentence (*r* = 0.19, *p* < .001), whereas negatively correlated with local and global arguments (*r*(local) = −0.27, *p* < .001; *r*(global) = −0.25, *p* = .002), connectives (*r* = −0.17, *p* = .028) and causal connectives (*r* = −0.27, *p* < .001). In addition, voice was negatively connected with local noun (*r* = −0.22, *p* = .005) and local stem (*r* = −0.17, *p* = .034), while positively related to global argument (*r* = 0.23, *p* = .002) and LSA all sentence (*r* = 0.27, *p* < .001). By contrast, in Chinese continuation, the cohesive variables presented no significant correlation with creative variables.

## Discussion

The present study adopted Coh-Metrix, CTAP, LIWC-22, and CLIWC-22 to analyze continuation writing by four LLMs (i.e., GPT-3.5, GPT-4, ERNIE, and SPARK) with a focus on cohesion and creativity. Employing both English and Chinese SWCTs, this study is dedicated to addressing three questions, namely the differences in writing productivity from the perspectives of cohesion and creativity, the correlations between creativity and cohesion in the LLM-generated continuations, and the discrepancy of linguistic competencies manifested in the continuations by LLMs.

The results indicated that in English continuation writing, ERNIE and SPARK performed the best in overall performance (i.e., narrativity, syntactic simplicity, and word concreteness) and cohesion (i.e., referential cohesion, deep cohesion, and latent semantic analysis), whereas GPT-4 exhibited the worst performance. As a whole, ERNIE and SPARK excelled in local cohesion, while GPT-3.5 and ERNIE displayed better global cohesion than SPARK and GPT-4 (sequenced according to scores). In contrast, creativity analysis revealed GPT-4 as the strongest, followed by GPT-3.5, with ERNIE failing to achieve satisfactory performance.

In Chinese continuation writing, ERNIE produced the most complex text with the highest number of words, phrases, and various character types, while GPT-4 generated the least, indicating lower complexity. With regard to local cohesion, GPT-3.5 manifested poorer performances than ERNIE and SPARK, with GPT-4 scoring the lowest in all local cohesion indexes. However, GPT-4 excelled in global cohesion. In the aspect of creativity, ERNIE showed the most robust generativity, slightly outperforming the other three LLMs. But statistically, the four LLMs showed no remarkable discrepancy in creativity in Chinese continuation generation. These results unveil the LLMs’ ability to construct coherent and creative English and Chinese continuations, hence answering the first question.

The correlation between cohesion and creativity revealed a significant relationship between cohesive variables, especially noun overlap, argument overlap, connectives, and LSA with creativity in English continuation. More specifically, noun overlap exhibited positive correlations with image, while showing positive correlations with voice. Arguments were negatively associated with image, and LSA all sentences were positively related to both image and voice. On the contrary, no significant association was identified between cohesion and creativity in Chinese continuation. These findings addressed the second research question.

The above results also suggest that the four LLMs displayed different formal linguistic competence across English and C-dominant LLMs. More specifically, GPT-4 dominated in cohesion in Chinese continuation and creativity in both English and Chinese continuations, whereas C-dominant LLMs excelled in cohesion in English continuation. Accordingly, it is largely rational to conclude that GPT-4 prevails in writing productivity. The following discusses why ChatGPTs and C-dominant LLMs displayed different writing performances.

In summary, this section is dedicated to the comparison between ChatGPTs and C-dominant LLMs in terms of cohesion and creativity in English and Chinese continuations, and to the correlation between cohesion and creativity (e.g., image and voice). Table 10 presents the results of the comparisons.

**Table 10:** Results of comparisons.

Measure Types		Indexes	Results
Overall Performance	Readability (English)	Narrativity (NA), syntactic simplicity (SS), word concreteness (WC)	C > GPT (NA) GPT > C (WC)
	Complexity	Different types of words and characters count	GPT > C (English) C > GPT (Chinese)
English Cohesion	Referential cohesion	Local and global noun/argument/stem overlap	C > GPT
	Deep cohesion	Connectives & causal connectives	C > GPT
	Latent semantic analysis (LSA)	LSA adjacent sentence, LSA sentence all	GPT > C
Chinese Cohesion	Referential cohesion	Local and global noun/word/lexical overlap	GPT > C
	Deep cohesion	Connectives	GPT > C
English Creativity	Image	Insight, feel, body	GPT > C
	Voice	All punctuations, comma, authenticity, dictionary word	GPT > C
Chinese Creativity	Image	Insight, feel,body	C > GPT
	Voice	All punctuations, informal language, dictionary word	GPT > C

* GPT: ChatGPTs, C: C-dominant LLMs, > : outperform

### ChatGPTs and C-dominant LLMs’ different performances in SCWT

The statistical analysis indicated that ChatGPTs and C-dominant LLMs displayed respective advantages and disadvantages in English and Chinese continuations. The following part is to discuss their different performances and possible reasons.

#### Complexity: GPTs > C-dominant LLMs in English continuation vs. C-dominant LLMs > GPTs in Chinese continuation.

ChatGPTs generated English continuations featuring more intricate sentence structures, whereas C-dominant LLMs’ Chinese continuations exhibited greater complexity. For English continuation, based on the word counting of continuations produced by the LLMs, GPTs were found to generate more big English words and longer sentences, contributing to higher reading difficulties. Adding to this vein are GPT-3.5 and GPT-4’s lower performances in narrativity for English continuation, suggesting lower readability.

This result may arise from the GPTs’ overuse of advanced and abstract English vocabulary, such as *iridescent*, *serenity*, and *bestow* by GPTs. In contrast, C-dominant LLMs were inclined to utilize more plain and straightforward words (e.g., *brightly colored* by ERNIE*,* and *make a big difference”* by SPARK). This phenomenon corresponds with Zhou et al. [5] that higher narrativity is related to the use of “familiar and easy-to-understand language”.

Further, another reason leading to GPTs’ higher writing complexity and weaker narrativity in English continuation is their lower utility of pronouns. Overuse of the first-person pronoun “I”, as noted by Li [59], can reduce formality while increasing narrativity. However, according to Petchprasert [60], utilizing the first-person pronoun was regarded as the writer’s effort to show their identity. In other words, employment of pronouns, especially the first-person pronouns, may create a conversation-like phenomenon, which may immerse readers in the texts. Therefore, GPTs’ minimal use of this pronoun may result in a disconnect between the author and the plot. In contrast, ERNIE and SPARK’s higher use of first-person pronouns creates a more engaging personal narrative.

C-dominant LLMs generated Chinese continuation with higher complexity. This can be seen in their use of more total words, multi-character phrases, and intermediate-stroke characters. ERNIE and SPARK generated an average of more total words, more three-character and two-character phrases, and more intermediate-stroke characters. Meanwhile, GPT-4 showed the least, with fewer words, phrases, sentences, and intermediate stroke characters. We deem that this complexity is very likely due to the C-dominant LLMs’ development in a Chinese environment with larger Chinese corpora, resulting in better Chinese language competence. Additionally, being more accessible to Chinese users and receiving mostly Chinese prompts contributed to their stronger performance in Chinese and slower progress in English.

#### Referential cohesion: C-dominant LLMs > GPTs for English continuation vs. GPTs > C-dominant LLMs for Chinese continuation.

C-dominant LLMs exhibited overall better performance in referential cohesion in English continuation than ChatGPTs. In English continuation, GPT-4 obtained the worst performance regarding referential cohesion, particularly in global and local cohesion (e.g., noun, argument, and stem overlap). The referential cohesion is closely related to word overlap [37], which scarcely occurs in GPT-4’s continuation, especially argument overlap. In addition, GPT-4’s continuations often reflected on events without advancing the author’s actions as shown in (a). C-dominant LLMs emphasized the protagonist’s next actions following the event in (b), (c), and (d), aligning more with the style of the preceding text and demonstrating stronger referential cohesion.

One possible reason for this difference may result from their discrepant frameworks, underlying architectures, and training corpora, leading to the diverse features in the texts [14,15,61]. Another resulting factor could be the evaluation metrics set by teachers in China to assess students’ English writing, which usually emphasizes the use of pronouns and connectives to show their logic. Adopting such writing texts as the pre-training corpus, C-dominant LLMs could be able to develop similar writing habits.

(a) *The encounter had subtly reminded me of*… and *the memory became a source of warmth and wonderment, reminding me of*… (GPT-4)

(b) *The next day, I visited a local animal rescue center to learn more about hummingbirds.* (ERNIE)

(c) *As I was sitting in my garden, I heard a familiar buzzing sound.* (GPT-3.5)

(d) *I decided to volunteer at a local animal shelter to help those who were less fortunate.* (SPARK)

GPTs performed better in referential cohesion in Chinese continuation. The results showed that GPTs gained an average greater performance in global noun overlap, global and local word overlap, and lexical word overlap. In contrast, C-dominant LLMs showed fewer repetitions of key terms, such as “一百元” (100 yuan), with GPT repeating it 11 times, GPT-4 26 times, ERNIE twice, and SPARK not at all. To our knowledge, this difference is due to the unique features of Chinese texts, which rely more on implicit cohesive devices, like topic chains, rather than explicit markers [62]. As a result, C-dominant LLMs, trained with more Chinese corpora, have a high possibility of generating Chinese texts with such features.

#### Deep cohesion: C-dominant LLMs > GPTs in English continuation vs. GPTs > C-dominant LLMs in Chinese continuation.

Our data showed that C-dominant LLMs used more conjunctions in English continuation. The results are supported by more frequent deep cohesion (i.e., connectives and causal connectives) markers used by ERNIE and SPARK than by GPTs. One possible reason for such results may be their pretraining datasets. ERNIE and SPARK are trained primarily on Chinese datasets, while GPTs are more English-based [14,15,61]. Consequently, trained with these datasets, C-dominant LLMs reflect writing styles similar to Chinese natives, while GPTs’ texts may contain writing styles consistent with English natives because LLMs can “resemble human language-selective network” [7]. Specifically, Chinese natives tend to overuse conjunctions in English writing, a habit encouraged in Chinese students’ writing practice by teachers [63,64].

GPT-3.5 outperformed the other three LLMs in connective usage in Chinese continuations by a small margin. However, connectives found in the continuations generated by the four LLMs were not as many as expected. This is because Chinese is a paratactic language, conveying grammatical meaning and logical relationships through phrases and words, instead of conjunctions, unlike English [65]. Another reason why Chinese conjunctions were identified much less than their English counterparts may be attributed to the conjunction corpus used in CTAP. CTAP is a newly developed program where the words in the corpus may not be adequate. Given the small number of conjunctions identified, GPT-3.5’s use of conjunctions was not excessive. Overall, GPTs demonstrated better application of conjunctions to create deep cohesion in Chinese writing.

According to literature, we infer that the different performances in deep cohesion relate to their embedding dimensions and layers. As indicated in Eldan and Li [66], the increase of embedding dimensions and the number of layers improves the model’s performance in text coherence and cause-effect relationship. Additionally, the embedding dimensions were responsible for grabbing word relations and layers for text coherence [66]. Therefore, with many more layers and parameters embedded in the architecture, GPTs were able to beat C-dominant LLMs in terms of deep cohesion.

#### Latent semantic analysis: ERNIE < GPTs.

Compared with other LLMs, ERNIE struggled with semantic cohesion in English continuations. Latent semantic analysis (LSA) measures semantic reoccurrences, such as “synonyms, antonyms, hyponyms, compounds”, between sentences or paragraphs [67]. The combination of both the index and plot suggests that the two GPTs focused on hummingbird encounters, using varied descriptions and denotations of the hummingbird and encounter. For example, one sample of GPT-4 produced a wide range of phrases related to the bird, as in (e) and their encounter, as in (f). Similarly, GPT-3.5 frequently repeated words from the source text, such as “hummingbird”, in nearly every paragraph. Further, GPT-3.5 focused its plot on the hummingbird, including its successive movement after the encounter. For example, the new project proposed in (g) centered around the hummingbird, the farm, and the protagonist’s friends. On the contrary, ERNIE introduced unrelated elements like planting native plants and reducing carbon footprints, diverging from the original plot and scoring lower in LSA for adjacent and all sentences.

(e) *her tiny wings, the hummingbird’s bright eyes and miraculous return, the hummingbird’s goodbye.* (GPT-4)

(f) *the journey, fleeting moments of interaction, this encounter, a reminder*. (GPT-4)

(g) *Inspired by the hummingbird’s resilience, I decided to take on a new project*. (GPT-3.5)

Distinct pre-training strategies are supposed to account for the difference between ERNIE and the other LLMs as well. GPTs use self-attention to encode input sequences and produce hidden representations, enhancing their ability to capture long-range dependencies and relationships at various abstraction levels [9]. On the contrary, ERNIE adopted a new “Continual Multi-Paradigms Unified Pre-training Framework” [15], which disconnects its top layers from the lower layers, allowing independent processing of task-specific features. These differing pre-training mechanisms may affect how they calculate cosine values between vectors, influencing latent semantic analysis [67].

#### Creativity: GPTs > C-dominant LLMs.

The statistical data revealed that GPTs excelled in C-dominant LLMs in both image and voice in English continuation. More specifically, GPTs excelled in feel and body, demonstrating stronger creativity in the image category, despite scoring lower in insight, which is negatively correlated with image [40]. In voice, GPTs surpassed ERNIE and SPARK in most variables, including dictionary words, which are also negatively correlated with voice [40]. Accordingly, it seems safe to conclude that GPTs are superior to C-dominant LLMs in English creativity.

However, ERNIE outperformed GPTs in the image, whereas GPT-4 surpassed C-dominant LLMs in voice in Chinese continuations by a narrow margin. The advantages of ERNIE in the image over the other LLMs manifested in the insight and the overall performance, where ERNIE gained better scores than the GPTs. As for voice, GPT-4 excelled in all punctuation, dictionary words, and overall performance of voice.

These findings delineate an impression wherein GPTs are better at depicting vivid mental images in English. “Image” in creative writing involves using concrete details, descriptions, and rhetorical devices to evoke readers’ senses and draw them into the characters’ minds, avoiding abstraction, generalization, and judgment [40,68]. This echoes the results of word concreteness, which demonstrated that GPTs employed more concrete words than C-dominant LLMs in English continuations. In addition, this difference also lies in the details of the description as shown in Examples (h), (i), (j), and (k). When describing a similar plot of recalling the struggling scene of the hummingbird in the webs, GPTs and ERNIE adopted specific verbs, adjectives, and substances, whereas SPARK used more abstract words, like *helpless* and *freedom*.

(h) *That hummingbird had been trapped, covered in spider-webs, struggling to escape, and I had been the one to set her free.* (GPT-3.5)

(i) *Driving back home, the hummingbird’s bright eyes and swift yet tender movements lingered in my mind.* (GPT-4)

(j) *I replayed the scene in my mind, the bird’s sticky webs and determined expression, her hovering and searching gaze.* (ERNIE)

(k) *The bird had been trapped and helpless, but with my assistance, she regained her freedom.* (SPARK)

ERNIE won in image in Chinese continuation for its continuation contained more details. However, in the Chinese continuation, the coding results were much lower than those in the English continuation. This is because compared to the English task, the Chinese task contains more characters needing detailed descriptions. Nonetheless, due to the word limit, the four LLMs depicted the three monkeys’ efforts more generally with little detail, presenting causes and effects instead of the detailed methods. Thus, it is worth noting that ERNIE provided details in some pieces, as shown in Example (l), leading to its highest score in the image.

(l) *他的故事从天上的星星到地上的蚂蚁，从森林里的野兽到海里的鱼虾，千奇百怪，无奇不有。 (His story ranges from the stars in the sky to the ants on the ground, from the beasts in the forest to the fish and shrimps in the sea, full of wonders and surprises.*) (by ERNIE)

Furthermore, the results also suggested that GPTs excelled in voice in continuations in both languages. The voice examined the extent to which one could form their unique writing styles [68]. Accordingly, using higher amounts of punctuation, commas, and fewer dictionary words in the existing corpus, GPT-4 gained superiority in voice, indicating its stunning amounts of vocabulary. However, GPT-4 scored the lowest in authenticity, which is represented by the usage of “first-person words and present-tense verbs” [40]. This result coincides with the results of narrativity that GPTs used less first-person than C-dominant LLMs.

#### Correlation between cohesion and creativity.

The results indicated that image correlated with referential cohesion, deep cohesion, and global LSA, while voice was related to referential cohesion and global LSA in English continuation. This is echoed by Zedelius et al. [40], who found that referential and deep cohesion had a negative effect on image and voice. Different from their research, this study reveals that argument overlap and connectives are negative predictors of image, whereas noun and stem overlaps are negatively linked to voice, extending Zedelius et al.[40]. Here, the negative relationship was predictable in that less coherent text requires longer time and more effort to infer and understand the text [69], which is deemed as one manifestation of creativity [40].

The positive correlation between voice and argument overlap may result from the reference of pronouns across sentences. The argument overlap examines the matching nouns and pronouns between two sentences [70]. While noun overlap negatively impacted voice, pronoun overlap likely had a positive effect, with pronouns, especially first-person ones, reflecting author identity [71,72]. Frequent pronoun use aids readers in constructing this identity [73], enhancing voice.

The enhancing effect of noun overlap on image is owing to the enhancement of details by noun overlaps. The noun overlap refers to the identical reference of nouns without morphological deviation across sentences [70]. The frequent reference of nouns or noun phrases may reduce ambiguity and increase plot concreteness [70], which aligns with the idea that image is tied to detail [68].

The positive influence of LSA-all-sentence on higher creativity (including image and voice) is predictable. The LSA-all-sentence calculates the similarity between sentences with all sentences considered, not merely adjacent sentences [69]. Previous research has applied LSA to measure creativity in the text or responses [74,75], suggesting the positive relationship between LSA and creativity. Further, creative thinking also refers to the innovative combination of distant or weak elements, and the greater the elements are, the more creative the outcome [76]. With this background, it is reasonable to infer that the ability to associate the distant semantic elements, i.e., LSA, would have a positive correlation with creativity.

By contrast, no correlation was found between cohesion and creativity shown in Chinese continuation. One reason may be the adaptability of the computational tools for Chinese texts. For instance, despite many punctuations used in Chinese continuations, the CLIWC cannot fully identify and code them. Another reason may result from the corpus used in the computational tools. The three NLP tools for coding may have adopted different Chinese corpora. As a result, texts parsed by NLPIR-Parser may not be acceptable by CTAP and CLIWC, leading to NLPIR-Parser may not be acceptable by CTAP and CLIWC.

### Formal linguistic competence

To begin with, the results support Mahowald et al. [7] that LLMs have been equipped with formal linguistic competence. Firstly, the writing generated by the four LLMs maintains good coherence with the previous section by deploying *it* and *doing so* to refer to the pre-mentioned plot as in Example (m) and complicated clauses and connectives to advance the plot as in Example (n). This suggests that all the LLMs have a deft command of syntax and grammar. Furthermore, our results provided evidence that the LLMs have learned hierarchical structures for long-distance agreements. For example, in Example (o), *hummingbird* and *had brought* were connected with correct grammar even though a manner-adverbial, *with her vibrant features*, was inserted.

(m) *It had been a moment of desperation, a moment when I had stepped in to save a life, and in doing so*. (GPT-3.5); *It was an indelible reminder of how even the smallest act of kindness can create a lasting impression*. (GPT-4); *and in doing so, we had formed a bond that transcended species.* (SPARK); *I recounted my hummingbird story and the impact it had on my life*. (ERNIE).

(n) *We marveled at the mysterious ways in which nature can touch our lives.* (GPT-3.5); *There was a bond, however fleeting, that had formed between us.* (GPT-4); *And although I missed her dearly, I understood that she …* (SPARK); *our actions can have profound consequences for others, even if those actions seem small*. (ERNIE)

(o) *The little hummingbird, with her vibrant feathers, had brought a miraculous touch to our simple cookout, turning it into a moment of awe and wonder.* (GPT-4)

More specifically, our data revealed that GPTs were more excellent in applying English words and syntax, namely possessing better formal linguistics in terms of English continuation tasks. For one thing, GPTs outperformed ERNIE and SPARK in producing complicated sentences and text with greater reading difficulty, indicating GPT’s superior syntactic ability [69]. For the other, GPTs were found to adopt more concrete words and creativity-related terms, along with better performance in latent semantic analysis, reflecting their larger corpus of synonyms, antonyms, and related word categories [69].

Nevertheless, C-dominant LLMs slightly surpassed GPTs in Chinese continuation. Firstly, the four LLMs generated sentences of similar length (GPT-3.5: 13.7 characters, GPT-4: 13.8 characters, ERNIE: 13.9 characters, SPARK: 13.8 characters), suggesting comparable syntactic complexity [69]. Second, GPTs and C-dominant LLMs had their advantages in different aspects of creativity in Chinese continuation (GPTs in voice while C-dominant LLMs in image). Additionally, C-dominant LLMs produced more complex sentences, characterized by intricate characters and phrases.

We believe that dissimilar pretraining datasets can partly explain the different syntactic and lexical performance across English and Chinese continuations as well. According to OpenAI [14], GPTs were trained depending on various databases and corpora, including English and Chinese. On the contrary, the corpus to train C-dominant LLMs focuses on Chinese datasets and under a Chinese environment [15]. Therefore, GPTs are able to obtain more English vocabulary than an equivalent Chinese vocabulary with C-dominant LLMs.

### Implications and limitations

This study provides significant insights into the adoption of LLMs in the writing practice, especially in the continuation writing teaching. Firstly, the results indicated that different LLMs demonstrated distinct strengths and weaknesses in terms of cohesion and creativity, suggesting that these LLMs may be applicable to provide specific and actionable feedback regarding the two aspects. Therefore, by utilizing LLMs for personalized feedback, teachers can offer tailored suggestions on individual student writing to improve the cohesion and creativity, particularly in bilingual SCWTs that involve complex narrative development.

Secondly, LLMs demonstrate strengths in creativity based on language context (e.g., GPT models perform better in generating imagery for English, while C-dominant models excel in creativity for Chinese). Writing teachers can leverage these strengths to guide students toward more imaginative and expressive writing. By adopting the LLMs in the feedback providing process on the basis of their respective strengths, students can learn how to craft vivid and engaging narratives. For example, given the strengths of depicting vivid images in English by ChatGPTs, they could be adopted to provide feedback on such kind during English narrative composition.

Further, the differences in cohesion and creativity performance across English and Chinese SCWTs highlight the importance of bilingual awareness in writing. Teachers can incorporate LLMs into exercises that compare writing in both languages, encouraging students to explore how cohesion and creativity function differently in each with the help of LLMs. This not only improves their writing in the target language but also enhances their ability to transfer writing skills between languages. Such practice encourages a deeper understanding of the structural and stylistic features of each language, fostering bilingual writing proficiency.

More specifically, for cohesive improvements in English narrative tasks, teachers and students can adopt C-dominant LLMs to provide suggestions on conjunction choices. As for the enhancement of creativity, i.e., for more vivid descriptions of actions and plots, teachers and students could leverage GPTs to offer advice on how to construct details concerning characters’ insights, feelings, and actions. According to our own practice, this combination of two different models does improve writing more satisfactorily relative to any single model, eastern or western.

Future research should expand along two main directions. First, this study focused exclusively on linguistic competence while neglecting cultural factors. A combined analysis of linguistic and cultural aspects would enhance the adaptability of LLMs across different languages and cultural contexts. Second, while this study assessed narrative and story writing based on cohesion and creativity, these findings may not generalize to other genres, such as argumentative or academic writing, which warrants further investigation. Lastly, NLP tools may rely on language-specific implementations, potentially influencing the results conducted in English and Chinese. Future research could address this limitation by developing or adopting cross-linguistically aligned NLP tools and evaluation pipelines, thereby improving the comparability of analyses across English and Chinese.

## Conclusion

The present study conducted a comparative evaluation of the writing performance by four large language models (e.g.,GPT-3.5, GPT-4, ERNIE, and SPARK) using English and Chinese Story Continuation Writing Tasks (SCWTs), with the focus on cohesion and creativity. The findings revealed that GPT-4 exhibited the highest overall writing productivity, while LLMs pretrained with dominant Chinese Corpus (C-dominant) excelled in referential and deep cohesion for English tasks. In contrast, GPT models demonstrated superior performance in LSA (latent semantic analysis) across English continuation tasks and for all subcategories of Chinese continuation tasks. In terms of creativity, GPT models excelled in image within English continuations and in voice for both English and Chinese continuations, whereas C-dominant LLMs outshone in generating image in Chinese continuations. Ultimately, the study underscores the greater formal linguistic competence of GPT models in English, while C-dominant LLMs displayed a slight advantage in Chinese linguistic tasks.

## Supporting information

S1 File The description of assessing indices (adopted from Coh-Metrix Version 3.0 Indices, n.d.; Kim et al., 2012; Petchprasert, 2021; Tausczik & Pennebaker, 2010). (DOC)

S2 File Prompts and input texts. (DOC)

S3 File Significant data results for overall performance, cohesion, and creativity. (DOC)

S4 File Coding data. (XLSX)
