# Supplementary material for: Writing creativity, cohesion, and formal linguistic competence in LLMs: A comparative evaluation based on English and Chinese continuation writing
Source: PLoS One. 2026 Jun 22;21(6):e0335185. doi: 10.1371/journal.pone.0335185 (PMC13286153; doi:10.1371/journal.pone.0335185)
Supplement: S1 File — (DOC) [file pone.0335185.s001.doc]

**S1 Table**. **The Description of Assessing Indexes** (adopted from Coh-Metrix Version 3.0 Indices, n.d.; Kim et al., 2012; Petchprasert, 2021; Tausczik & Pennebaker, 2010)

| Discourse components | | Variables | Description |
| --- | --- | --- | --- |
|  | Narrativity | / | The text tells a story with characters, events, places, and things that are familiar to a reader. Stories are basically about everyday oral conversation. |
|  | Syntactic Simplicity | / | This component shows the degree to which the sentences in the text contain fewer or more words with the uses of simple, familiar or complex, unfamiliar syntactic structures. |
|  | Word Concreteness | / | Content words are concrete, meaningful, and simple to understand. Abstract words are difficult to represent visually, so the texts that have a lot of abstract words tend to be more challenging than those content words. |
| Cohesion | Noun Overlap | Local & Global | noun overlap represents the average number of sentences in the text that have noun overlap from one sentence back to the previous sentence. Local overlap considers only adjacent sentences, global overlap considers the overlap of each sentence with every other sentence |
|  | Argument Overlap | Local & Global | Argument overlap occurs when there is overlap between a noun in one sentence and the same noun in another sentence; it also occurs when there are matching personal pronouns between two sentences. |
|  | Stem Overlap | Local & Global | These two local and global overlap measures relax the noun constraint held by the noun and argument overlap measures. A noun in one sentence is matched with a content word in a previous sentence that shares a common lemma. |
|  | Latent Semantic Analysis | Sentence & All Sentence | this index provides measures of semantic overlap between sentences or between paragraphs |
|  | Connectives | All Connectives & Causal Connectives | This component assesses the use of connectives |
| Discourse components | | Variables | Sample Words |
| Creativity | Image | Insight | think, know, consider |
|  |  | Feel | feels, touch |
|  |  | Body | cheek, hands (useful to describe feelings and bodily sensations) |
|  | Voice | Authenticity |  |
|  |  | Informal Language | assents, fillers, swear words, netspeak |
|  |  | Dictionary | words in the LIWC 2015 dictionary file |
|  |  | All Punctuations | periods, commas, etc. |
|  |  | Comma |  |
