# Supplementary material for: Writing creativity, cohesion, and formal linguistic competence in LLMs: A comparative evaluation based on English and Chinese continuation writing
Source: PLoS One. 2026 Jun 22;21(6):e0335185. doi: 10.1371/journal.pone.0335185 (PMC13286153; doi:10.1371/journal.pone.0335185)
Supplement: S2 File — (DOC) [file pone.0335185.s002.doc]

**S2 File. Prompts and input texts**

SCWT:

1. English SCWT

*“阅读以下材料，根据其内容和所给段落续写一篇短文补充结局，字数为150左右” (Please read the following text and complement an English ending according to the given context with a word limit of around 150.)*

I was invited to a cookout on an old friend's farm in western Washington. I parked my car outside the farm and walked past a milking house which had apparently not been used in many years. A noise at a window caught my attention, so I entered it. It was a hummingbird, desperately trying to escape. She was covered in spider-webs and was barely able to move her wings. She ceased her struggle the instant I picked her up.

With the bird in my cupped hand, I looked around to see how she had gotten in. The broken window glass was the likely answer. I stuffed a piece of cloth into the hole and took her outside, closing the door securely behind me.

When I opened my hand, the bird did not fly away; she sat looking at me with her bright eyes. I removed the sticky spider-webs that covered her head and wings. Still, she made no attempt to fly. Perhaps she had been struggling against the window too long and was too tired? Or too thirsty?

As I carried her up the blackberry-lined path toward my car where I kept a water bottle, she began to move. I stopped, and she soon took wing but did not immediately fly away. Hovering, she approached within six inches of my face. For a long moment, this tiny creature looked into my eyes, turning her head from side to side. Then she flew quickly out of sight.

During the cookout, I told my hosts about the hummingbird incident. They promised to fix the window. As I was departing, my friends walked me to my car. I was standing by the car when a hummingbird flew to the center of our group and began hovering. She turned from person to person until she came to me. She again looked directly into my eyes, then let out a squeaking call and was gone. For a moment, all were speechless. Then someone said," She must have come to say good-bye."

1. Chinese SCWT:

*“阅读以下材料，根据其内容和所给段落续写一篇短文补充结局，字数为250左右” (Please read the following text and complement a Chinese ending according to the given context with a word limit of around 250.)*

在很久很久以前，海边的大森林里住着猴妈妈一家人。猴妈妈有三个孩子，老大叫猴听话，老实本分，诚实正直;老二叫猴机灵，聪明伶俐，爱动脑筋;老三是人女孩，叫猴美丽，长得很漂亮，也很可爱。因为猴妈妈很勤劳，所以他们一家人的日子过得挺好的，不愁吃，不愁穿家人快乐地生活着。

猴妈妈一家祖祖辈辈都生活在这片大森林里，所以，三个小猴子从出生到现在也一直住在这里，从来没有走出去过，没有见过森林的那边是什么。因为这样，他们三个对外面的世界充满了好奇，特别想走出这片大森林，去看看外面的世界。猴妈妈十分明白自己的孩子在想什么她也想让三个孩子出去见见世面，开阔开阔眼界。于是，她做了一个重要的决定:让孩子去外面生活。

这天，猴妈妈做了很多好吃的，小猴子们吃得很开心。吃完饭后，猴妈妈把三个孩子叫到跟前，握着他们的手，对他们说了很多话，然后给他们每个人发了100元钱，让他们离开家，去外面的世界独立生活，看看谁能够过上富足的日子。

三个孩子听了之后，非常高兴，终于可以离开这片森林了。他们回到自己的房间，很快就收拾好自己的东西，然后一个一个去跟猴妈妈道别。之后，他们各自拿着自己的钱，带上自己的行李，离开了这片大森林，去独立生活了。
