# Supplementary material for: Writing creativity, cohesion, and formal linguistic competence in LLMs: A comparative evaluation based on English and Chinese continuation writing
Source: PLoS One. 2026 Jun 22;21(6):e0335185. doi: 10.1371/journal.pone.0335185 (PMC13286153; doi:10.1371/journal.pone.0335185)
Supplement: S3 File — (DOC) [file pone.0335185.s003.doc]

**S3 File. Significant data results for overall performance, cohesion, and creativity**

**Overall Performance in English Continuation**

Narrativity

*β*(GPT-3.5 – GPT-4) = 37.43, *p* < .000

*β*(GPT-3.5 - ERNIE) = -14.88, *p* < .000

*β*(GPT-3.5 - SPARK) = -13.84, *p* < .000

*β*(GPT-4 - ERNIE) = -52.32, *p* < .000

*β*(GPT-4 - SPARK) = -51.27, *p* < .000

word correctness

*β*(GPT-3.5 - ERNIE) = 13.75, *p* = 0.0258;

*β*(GPT-4 - ERNIE) = 17.74, *p* = .0069

*β(*ERNIE - SPARK) = -14.30, *p* = .0154

**Cohesion in English Continuation:**

local noun overlap:

*β*(SPARK – GPT-3.5) = -0.055, *p* = .0343;

*β*(SPARK – GPT-4) = -0.1052, *p* = .0067;

*β*(SPARK – ERNIE) = -0.071, *p* = .041

local argument overlap:

*β*(GPT-3.5 – ERNIE) = -0.202, *p* = <.0001;

*β*(GPT-4 – ERNIE) = -0.294, *p* <.0001

*β*(GPT-3.5 – SPARK) = -0.151, *p* = .002;

*β*(GPT-4 – SPARK) = -0.242, *p* <.0001

connectives:

*β*(GPT-3.5 – GPT-4) = 18.40, *p* < .0001

*β*(GPT-3.5 – SPARK) = -8.686, *p* = .0217

*β*(GPT-4 – ERNIE) = -24.58, p < .0001;

*β*(GPT-4 – SPARK) = -27.09, *p* < .0001

causal connectives:

*β*(GPT-4 – ERNIE) = -10.06, *p* = . 0045;

*β*(GPT-4 – SPARK) = -14.31, *p* < .0001

*β*(GPT-3.5 – SPARK) = -8.783, *p* = .0196

global noun overlap

*β*(GPT-3.5 –GPT-4) = 0.015, *p* < .0001;

*β*(GPT-3.5 – ERNIE) =0.0128, *p* = .0041;

*β*(GPT-3.5 – SPARK) = 0.028, *p* < . 0001

*β*(GPT-4 – SPARK) = 0.012, *p* = .0001

*β*(SPARK – ERNIE) = -0.015, *p* = .0001

global argument overlap

*β*(GPT-4 – GPT-3.5) = -0.038, *p* < .0001

*β*(GPT-4 – ERNIE) = -0.046, *p* < .0001

*β*(GPT-4 – SPARK) = -0.049, *p* < .0001

global stem overlap

*β*(GPT-3.5 – GPT-4) = 0.021, *p* < .0001;

*β*(GPT-3.5 – ERNIE) = 0.014, *p* = .0354;

*β*(GPT-3.5 – SPARK) = 0.035, *p* < .000

*β*(ERNIE – SPARK) = 0.022, *p* < .0001

LSA adjacent sentences

*β*(GPT-4 – GPT-3.5) = -0.008, *p* = .0268

*β*(GPT-4 – ERNIE) = 0.0099, *p* =.0016

LSA all sentences

*β*(GPT-3.5 – GPT-4) = 0.006, *p* = .0045

*β*(GPT-3.5 – ERNIE) = 0.01, *p* < .0001

**Creativity in English Continuation**

Insight

*β*(SPARK-GPT3.5) = 1.0096, *p* = .001;

*β*(SPARK-GPT-4) = 0.767, p = 0.047;

*β*(SPARK-ERNIE) = 1.149, *p* < .001

feel

*β*(GPT-4 – ERNIE) = 1.004, *p* < .001

*β*(GPT-4 – SPARK) = 0.906, *p* < .001

*β*(GPT-3.5 – ERNIE) = 0.715, *p* < .001

*β*(GPT-3.5 – ERNIE) = 0.617, *p* < .001

body

*β*(GPT-4 – ERNIE) = 0.630, *p* < .001

*β*(GPT-4 – SPARK) = 0.537, *p* < .001

*β*(GPT-3.5 – ERNIE) = 0.337, *p* = .032

all punctuation

*β*(ERNIE – GPT-3.5) = -2.104, *p* < .001;

*β*(ERNIE – GPT-4) = -2.594, *p* < .001;

*β*(ERNIE - SPARK) = -1.582, *p* <.001

Comma

*β*(GPT-4 – GPT-3.5) = 1.546, *p* <.001;

*β*(GPT-4 – ERNIE) = 3.011, *p* <.001;

*β*(GPT-4 – SPARK) = 3.223, *p* < .001

*β*(GPT-3.5 – ERNIE) = 1.465, *p* <.001;

*β*(GPT-3.5 – SPARK) = 1.676, *p* <.001).

authenticity

*β*(GPT-4 – GPT-3.5) = -15.845, *p* = .001;

*β*(GPT-4 – SPARK) = -18.079, *p* < .001

dictionary

*β*(GPT-4 – GPT-3.5) = -5.086, *p* < .001;

*β*(GPT-4 – ERNIE) = -.5.082, *p* < .001;

*β*(GPT-4 – SPARK) = -.7.460, *p* < .001

*β*(SPARK – GPT-3.5) = 2.374, *p* < .001;

*β*(SPARK - ERNIE) = 2.378, *p* <.001

overall performance in “image”

*β*(GPT-3.5 – ERNIE) = 0.999, p = .029;

*β*(GPT-3.5 – SPARK) = 1.957, p < .001;

*β*(GPT-4 – ERNIE) = 1.252, p = .003;

*β*(GPT-4 – SPARK) = 2.210, p< .001

**Cohesion in Chinese Continuation:**

local lexical overlap

*β*(GPT-3.5 – ERNIE) = 0.077, *p* < .0090;

*β*(GPT-3.5 – SPARK) = 0.134, *p* < .0001

*β*(GPT-4 – SPARK) = 0.098, *p* < .0012

conjunction

*β*(GPT-3.5 – GPT-4) = 0.00984, *p* < .0001;

*β*(GPT-3.5 – ERNIE) = 0.00986, *p* < .0001;

*β*(GPT-3.5 – SPARK) = 0.00710, *p* = .0009

global noun overlap

*β*(ERNIE – GPT-3.5) = -0.0182, *p* = .0025;

*β*(ERNIE – GPT-4) = -0.0223, *p* = .0001;

*β*(ERNIE – SPARK) = -0.0227, *p* = .0077

global word overlap

*β*(GPT-3.5 – ERNIE) = 0.0175, *p* = .0049;

*β*(GPT-3.5 – SPARK) = 0.0122, *p* = .0487;

*β*(GPT-4 – ERNIE) = 0.0228, *p* = .0001;

*β*(GPT-4 – SPARK) = 0.0175, *p* = .0016

global lexical overlap

*β*(GPT-4 – ERNIE) = 0.0279, *p* < .0001;

*β*(GPT-4 – SPARK) = 0.0178, *p* = .0135

*β*(GPT-3.5 – ERNIE) = 0.0227, *p* = .0003

**Creativity in Chinese Continuation:**

dictionary

*β*(SPARK– GPT-4) = 2.332, *p* = .003

informal language

*β*(SPARK – GPT-3.5) = 0.924, *p* = .005;

*β*(SPARK – GPT-4) = 1.097, *p* < .001;

*β*(SPARK - ERNIE) = 1.156, *p* < .001

all punctuation

*β*(GPT-4– GPT-3.5) = 0.382, *p* <.001;

*β*(GPT-4 – SPARK) = 0.329, *p* = .003
